# Detection of methadone and buprenorphine in urine samples from inmates of German prisons

**DOI:** 10.3389/fpsyt.2023.1139370

**Published:** 2023-02-28

**Authors:** Giorgia Franchetti, Annette Thierauf-Emberger, Anette Franz, Vanessa Thoma, Volker Auwärter, Laura M. Huppertz

**Affiliations:** ^1^Department of Cardiac, Thoracic, Vascular Sciences and Public Health, Unit of Legal Medicine and Toxicology, University of Padova, Padova, Italy; ^2^Institute of Forensic Medicine, Forensic Toxicology, Medical Center, University of Freiburg, Freiburg, Germany

**Keywords:** prison, prescription drugs, methadone, buprenorphine, illicit use

## Abstract

The use of non-prescribed opioid substitution drugs is a serious public health problem, involving general population as well as vulnerable populations such as prisoners. The estimation of the prevalence of opioid substitution drug misuse in prisoners is crucial to suggest strategies to contrast this phenomenon and reduce the associated morbidity and mortality. The present study aimed to provide an objective estimation of the prevalence of illicit use of methadone and buprenorphine in two German prisons. Urine samples were collected from inmates of Freiburg and Offenburg prisons at random times and tested for the detection of methadone, buprenorphine and their metabolites. Analyses were performed by a validated liquid chromatography–tandem mass spectrometry (LC–MS/MS) method. In total 678 inmates participated in this study. The participation rate was about 60% of all permanent inmates. Of the 675 samples suitable for the analysis, 70 samples (10.4%) tested positive for methadone, 70 samples (10.4%) for buprenorphine, and 4 samples (0.6%) for both drugs. At least 100 samples (14.8%) were not associated with reported prescribed-opioid substitution treatment (OST). Buprenorphine was the most common illicitly used drug. In one of the prisons, buprenorphine was brought in from the outside. The present cross-sectional experimental study was able to provide reliable information regarding the illicit use of opioid substitution drugs in prisons.

## 1. Introduction

The rate of substance use disorder in prison population is significantly higher compared to the general population, with an estimated prevalence of 30–40% in German inmates (1–3).

Opioid use disorder in particular is overrepresented among prisoners (4). The main treatment options for opioid use disorder consist of abstinence programs and opioid maintenance treatment (5). For medical as well as ethical reasons, the implementation of opioid substitution treatment (OST) in prison settings is recommended by international guidelines in most European countries (6). Methadone and buprenorphine are two of the medications most frequently prescribed for OST (7). Both are opioids with effects on the central nervous system similar to those of morphine or heroine, thus preventing withdrawal symptoms and inhibiting the craving for opioid drugs, without promoting “high” conditions. It has been shown that OST is an effective treatment for opioid-dependent inmates, with benefits similar to those in community settings (3, 8).

On the other hand, despite the implementation of OST in prisons, it is well-known that prisoners use OST medications for illicit purposes during incarceration (9). Illicit use of methadone or buprenorphine is often related to self-medication purposes by opioid-dependent individuals who want to avoid withdrawal symptoms, perform self-detoxification, or prefer to manage substitution treatment on their own. However, OST medications can also be used for their euphoric effects in non-opioid dependent individuals, especially when combined with other substances (10).

There are several problems associated with the illicit use of drugs during incarceration. Among these, unpredictable clinical conditions resulting from intravenous administration of drugs of unknown quality and quantity, transmission of infectious diseases such as HCV and HIV due to needle sharing and/or reutilization, as well as increased risks for committing suicide (4). Furthermore, drug-overdose is one of the leading causes of fatality among prisoners during incarceration as well as the main cause of death after release from prison, with a significantly elevated risk up to at least 4 weeks following release (4, 11).

The estimation of the prevalence of opioid substitution drug misuse in prisoners is crucial to suggest public health interventions aimed at reducing the morbidity and mortality associated with this phenomenon. Moreover, such information can also be useful in the forensic field to investigate prisoners’ deaths. The available literature is scarce and the provided prevalence estimates are mainly based on questionnaires or clinical interviews (12–15). Therefore, the aim of the present study was to provide an objective estimation of the prevalence of illicit use of the two main opioid substitution drugs (i.e., methadone and buprenorphine) in two German prisons. This was carried out by analyzing urine samples collected from the inmates for methadone, its metabolite EDDP, buprenorphine, and its metabolite norbuprenorphine with liquid-chromatography tandem-mass spectrometry (LC–MS/MS).

## 2. Materials and methods

### 2.1. Experimental setup

This study builds on the research project concerning the detection of ethanol consumption markers in urine samples conducted in the same jails (16). The study was approved by the Ethics Committee of the University of Freiburg (project nr. 386/10).

In collaboration with the jails’ medical units, permanent inmates from the adult male prisons in Freiburg and Offenburg (Baden-Württemberg, Germany) were asked to provide a urine sample. Pre-trial inmates were not included. Participation was voluntary and written informed consent was given by each person included and tested. Each inmate was allowed to participate only once. The sampling took place at random times over a period of 12 months. Urine sampling was carried out in the presence of medical personnel to avoid any manipulation. All samples were anonymized.

The jails’ medical units informed the authors that both methadone and buprenorphine were available for OST in the Offenburg prison, while in the Freiburg prison only methadone was used. In the Offenburg prison in particular, for each collected sample, information on prescribed-OST drugs (i.e., methadone and buprenorphine) was provided. Such information was not provided in the Freiburg prison.

### 2.2. Determination of methadone, EDDP, buprenorphine, and Norbuprenorphine

#### 2.2.1. Chemicals and instrumentation

Certified reference standards of methadone, EDDP, buprenorphine, and norbuprenorphine, as well as their respective deuterated analoga (D9-methadone, D3-EDDP, D4-buprenorphine, and D3-norbubprenorphine) were obtained from Lipomed (Weil am Rhein, Germany) and LGC Standards GmbH (Wesel, Germany). Methanol was delivered by Sigma-Aldrich (Taufkirchen, Germany). Formic acid was obtained from AppliChem (Darmstadt, Germany), dicloromethane and 2-propanol were from Carl Roth GmbH & Co.KG (Karlsruhe, Germany). Ammonia (25%) and hydrochloric acid were purchased from Sigma (Steinheim, Germany), and acetic acid (0.1 M) was from Merck (Darmstadt, Germany). All solvents were of analytical or HPLC grade.

Analysis was performed with a liquid chromatography-electrospray ionization tandem mass spectrometry (LC-ESI-MS/MS) system consisting of a QTrap 2000 triple quadrupole mass spectrometer (ABSCIEX, Darmstadt, Germany) equipped with a TurboIonSpray™ interface, and an Agilient 1,100 series HPLC-system (Agilent, Waldbronn, Germany). Chromatographic separation was performed on a reversed-phase LC-column (Luna PFP, 150 × 2 mm, 4 μm, Phenomenex, Aschaffenburg, Germany) with matching guard column (4 mm x 2 mm, packing material identical; Phenomenex, Aschaffenburg, Germany) at 40°C, by a 15-min gradient elution using H2O with 1% formic acid and 0.1% ammonium formate (eluent A) and methanol with 1% formic acid (eluent B) as follows: 0 min, 95% A at 400 μL/min; 10 min, 2% A at 600 μL/min; 12.5 min, 95% A at 400 μL/min. A flow of 200 μL/min 2-propanol was added post column to enhance ionisation efficiency.

Data acquisition, evaluation, and quantitation was performed using Analyst® software including the software tool QuantitationWizard (V.1.5; AB SCIEX, Darmstadt, Germany). The MS was operated in positive electrospray ionization mode monitoring two transitions per analyte and one for each deuterated internal standard.

The calibration range was 1 to 250 ng/mL. The lowest calibration level corresponds to the lowest limit of quantitation (LLOQ) in urine, which was determined according to forensic guidelines. The limit of detection was 0.5 ng/mL.

#### 2.2.2. Sample preparation

Prior to solid phase extraction (SPE), 10 μL of internal standard-mix, containing D9-methadone, D3-EDDP, D4-buprenorphine, and D3-norbubprenorphine at a concentration of 200 ng/mL and 2 μg/mL respectively, were added to 1 mL of urine. For enzymatic hydrolysis, 50 μL ß-glucuronidase/sulfatase (helix promatia Type HP-2, Sigma-Aldrich, Steinheim, Germany) and 1 mL phosphorous buffer (pH6) were added. The samples were vortexed for 10 s and incubated at 45°C for 45 min. The sample volume was filled up to 3 mL with phosphorous buffer and vortexed. SPE was automated using an Aspec apparatus (Gilson, Middleton, WI, United States), Chromabond Drug Cartridges (200 mg, Macherey Nagel, Düren, Germany), and an opioid specific method (opiate 3 mL).

The extracted samples were transferred to LC vials and evaporated under a gentle stream of nitrogen at 40°C. The samples were reconstituted in 100 μL of LC Eluent A:B 95:5 (v/v).

For analysis, 10 μL were injected into the LC–MS/MS system.

Samples were found to be positive for the respective drug if either the parent compound and the metabolite or the respective metabolite only were detected.

### 2.3. Determination of creatinine

Creatinine was determined using the Jaffé reaction (DRI® Creatinine-Detect Test, Microgenics, Passau, Germany) on a Konelab 30i analyzer (Thermo Scientific, Dreieich, Germany).

## 3. Results

In total 678 urine samples were collected: 557 in Offenburg and 121 in Freiburg. The overall participation rate was about 60%: 70–75% of all permanent inmates in Offenburg and 30.6% of all permanent inmates in Freiburg participated. Creatinine determination in urine allowed to exclude two adulterated samples from the study, both from the Freiburg prison. One urine sample from the Offenburg prison was discarded due to insufficient volume. Therefore, 675 urine samples were suitable for the analysis, 556 from Offenburg and 119 from Freiburg.

**Figure 1:**
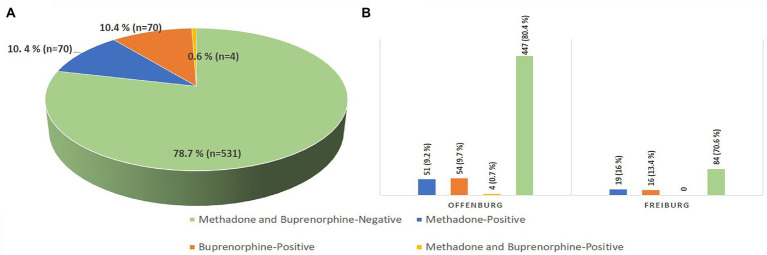
Results of the qualitative analysis of urine samples from the prison population investigated in total **(A)** and, separated by prison **(B)**. **(A)** Out of 675 tested samples, 70 samples (10.4%) tested positive for methadone, 70 samples (10.4%) tested positive for buprenorphine, 4 samples tested positive for both substances, and 531 samples (78.7%) tested negative for methadone and buprenorphine. **(B)** Out of the 556 samples tested in the Offenburg prison, 51 samples (9.2%) tested positive for methadone, 54 samples (9.7%) tested positive for buprenorphine, 4 samples (0.7%) tested positive for both substances and 447 samples (80.4%) tested negative for methadone and buprenorphine. Out of the 119 samples tested in the Freiburg prison, 19 samples (16%) tested positive for methadone, 16 samples (13.4%) tested positive for buprenorphine and 84 samples (70.6%) tested negative for methadone and buprenorphine.

A total of 144 urine samples (109 from Offenburg, 35 from Freiburg) were positive for the investigated OST medications: 70 samples for methadone (51 from Offenburg, 19 from Freiburg), 70 samples for buprenorphine (54 from Offenburg, 16 from Freiburg) and 4 samples for both substances (4 from Offenburg). The remaining 531 samples (447 from Offenburg, 84 from Freiburg) were negative for methadone and buprenorphine (Figure 1).

In the Offenburg prison, 25/109 positive samples were found in association with reported prescribed OST. The remaining 84/109 positive samples (30/84 for methadone, 50/84 for buprenorphine and 4/84 for methadone and buprenorphine) were not associated with prescribed OST. In the Freiburg prison, 16/35 positive samples (16/16 for buprenorphine) were not associated with prescribed OST. Due to the lack of information on the prescription of methadone for the samples collected in Freiburg, it was not possible to attribute the remaining 19/35 positive samples (19/19 for methadone) to prescribed or non-prescribed OST use. As a result, at least 100 positive samples (14.8%, out of 675 participants) were not associated with prescribed OST.

**Figure 2:**
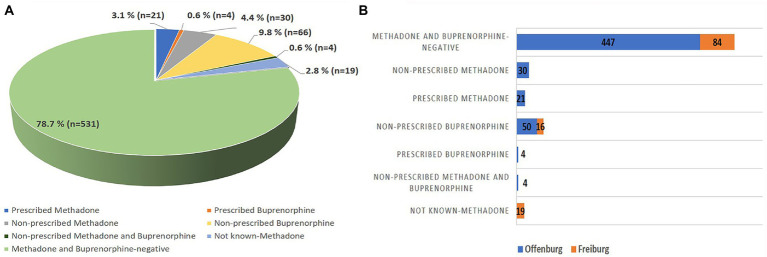
Type of OST drug use in the prison population investigated in total **(A)** and, separated by prison **(B)**. **(A)** Out of 675 tested samples, 21 samples (3.1%) were associated with prescribed-methadone, 30 samples (4.4%) were associated with non-prescribed methadone, 4 samples (0.6%) were associated with prescribed-buprenorphine, 66 samples (9.8%) were associated with non-prescribed buprenorphine, 4 samples (0.6%) were associated with non-prescribed combined use of methadone and buprenorphine, 19 samples (2.8%) were not attributable to prescribed or non-prescribed methadone, and 531 (78.7%) samples tested negative for methadone and buprenorphine. **(B)** Out of the 556 samples tested in the Offenburg prison, 21 samples (3.8%) were associated with prescribed-methadone, 30 samples (5.4%) were associated with non-prescribed methadone, 4 samples (0.7%) were associated with prescribed-buprenorphine, 50 samples (9%) were associated with non-prescribed buprenorphine, 4 samples (0.7%) were associated with non-prescribed combined use of methadone and buprenorphine, and 447 samples (80.4%) tested negative for methadone and buprenorphine. Out of the 119 samples tested in the Freiburg prison, 19 samples (16%) were not attributable to prescribed or non-prescribed methadone, 16 (13.4%) samples were associated with non-prescribed buprenorphine, 84 samples (70.6%) tested negative for methadone, and buprenorphine.

Detailed results regarding prevalence of OST drug detection, type of OST drug detected and type of OST drug use for the prison population investigated are shown in Table 1 and Figure 2.

**Table 1:** Prevalence of OST drug consumption, type of OST drug detected, and type of OST drug use in the prison population investigated.

	**Offenburg**	**Freiburg**	**Both prisons**
**Tested samples**	556	119	675
**Detection of OST drug**			
Positive	109 (19.6 %)	35 (29.4 %)	144 (21.3%)
Negative	447 (80.4 %)	84 (70.6 %)	531 (78.7%)
**OST drug detected**			
Methadone	51 (9.2 %)	19 (16 %)	70 (10.4%)
Buprenorphine	54 (9.7 %)	16 (13.4 %)	70 (10.4%)
Methadone and Buprenorphine	4 (0.7 %)	0	4 (0.6%)
**OST drug use**			
*Prescribed*	25 (4.5 %)	0 – 19^a^ (0 – 16%)	25 – 44^a^ (3.7 – 6.5 %)
Methadone	21 (3.8 %)	0 – 19^a^ (0 – 16%)	21 – 40^a^ (3.1 – 5.9 %)
Buprenorphine	4 (0.7 %)	0	4 (0.6%)
*Non-prescribed*	84 (15.1 %)	16 – 35^a^ (13.4 – 29.4 %)	100 – 119^a^ (14.8 – 17.6 %)
Methadone	30 (5.4 %)	0 – 19^a^ (0 – 16 %)	30 – 49^a^ (4.4 – 7.2 %)
Buprenorphine	50 (9 %)	16 (13.4%)	66 (9.8%)
Methadone and Buprenorphine	4 (0.7 %)	0	4 (0.6%)

a 19 methadone-positive samples from Freiburg prison were not attributable to prescribed or non-prescribed OST use.

## 4. Discussion

The consumption of OST drugs without prescription as well as of illicit drugs and ethanol is prohibited in jail; nevertheless it is a matter of fact that these substances are illicitly taken by incarcerated individuals (16–18).

While previous studies regarding the prevalence of non-prescribed OST drugs use in jails are predominately based on questionnaires or clinical interviews (12–15), this study provides more objective data, by performing toxicological analyses on urine samples from inmates of two German prisons. The detection of methadone or buprenorphine and their metabolites in urine samples indicates relatively recent consumption. Depending on the dose, frequency of use and individual metabolism, the performed tests allowed for the detection of methadone/EDDP and buprenorphine/norbuprenorphine for 72–144 h and 48–96 h, respectively, after the last intake (19–21). Information, whether OST drugs were prescribed in prison or not, allowed to attribute the detected substance to prescribed or non-prescribed use for most cases. Since the Offenburg prison reported 25 cases of prescribed-OST drugs among participants, the remaining 84 positive samples were attributed to non-prescribed use. Since in the Freiburg prison buprenorphine was not available in the OST program; the 16 buprenorphine-positive samples were attributed to non-prescribed use. The 19 methadone-positive samples from the Freiburg prison could not be attributed to prescribed or non-prescribed use, since the number of participants with prescribed OST medication was not available.

Hence, our results indicate that at least 14.8% of participating inmates (100 out of 675) illicitly used OST drugs in the prisons of Freiburg and Offenburg.

We believe that the prevalence rate of illicit use provided by our study might be underestimated, due to the voluntary participation. Obviously, inmates who illicitly used drugs were more likely to not participate in the study. Especially in the Freiburg prison, where the non-participation rate was higher, a higher prevalence rate of illicit use would be expected. Furthermore, it cannot be ruled out that subjects on prescribed-OST might use the prescribed drugs in an illicit way, for example by taking higher doses of the substance or through illicit routes of administration. Therefore, our results suggest that the illicit use in the two prisons investigated could be even more widespread than detected, in accordance with previous studies, which reported prevalence rates ranging from 17 to 70% (12, 13).

To ensure that the objective data we have provided is also informative, a high participation rate was required. The participation rate was 70–75% in Offenburg and 30.6% in Freiburg. In the authors’ opinion, the higher participation in Offenburg was achieved because the medical personnel asking for the urine sample collection worked in the medical department of the Offenburg prison. The prisoners in Offenburg therefore were more prone to believe in the purely scientific use of the samples, without worrying about forensic implications. However, the guarantee of anonymization of the samples ensured a comprehensively high participation and thus a good detectability of methadone and buprenorphine consumption.

Moreover, all urine samples were collected at a specific point in time; thus, the results can be regarded as a representative cross section of the participants.

Another relevant finding of the present study addresses the origin of illicitly used OST drugs. The literature suggests that the main sources of opioids in jails are the OST medications prescribed in prisons, through the circumvention of the monitoring process of supervised consumption. In our study buprenorphine was found in the urine samples of participants from the Freiburg prison, although its prescription was not included in the OST program. This study therefore revealed that the buprenorphine detected in those urine samples must have been brought in from outside. This result was consistent with a recent systematic review by Bi-Mohammed et al. (12), who found out that drugs were brought in prisons by individuals from the community, especially during prison visits or by throwing drugs from the outside over the prison wall. Drugs may also be detected in letter and cards sent to prisoners by mail, where the paper was found to be impregnated with liquids consisting of drugs dissolved in organic solvents and then dried (22).

Regarding the type of OST drug used by the inmates, our results indicate that both methadone and buprenorphine were illicitly used, by 30 and 66 study participants, respectively. A combined illicit use of both substances was found in 4 cases. Accordingly, buprenorphine was the most commonly illicitly used OST drug (70 vs. 34 participants). Even if assuming that all the 19 methadone-positive samples from the Freiburg prison, whose type of use (i.e., prescribed or non-prescribed) was unknown, were associated with non-prescribed use, the total number of participants who illicitly used methadone would be a maximum of 53. This finding seems to support a preference for buprenorphine by inmates, as reported by previous studies (12–14, 23). In particular, a recent systematic review highlighted changing trends on the prescription opioid abuse in the prison settings from 1995 to 2015, with early studies identifying a prevalence of methadone (although very low), whereas latter studies concurred regarding the high prevalence of buprenorphine (12). Furthermore, a descriptive survey commissioned by the UK Ministry of Justice on 139 prisons identified buprenorphine as the most misused drug in 11 prisons, and the third most misused drug overall (24). The higher prevalence of illicit use of buprenorphine might be explained by the fact that, although both methadone and buprenorphine, like other opioid drugs, have the potential for misuse, they exhibit some differences (25). Methadone acts as pure agonist on the μ-opioid-receptor and possibly the σ-opioid-receptor, while buprenorphine is a partial agonist with high affinity toward the μ-opioid-receptor and additionally exhibiting an agonist-partial agonist–antagonist pattern on different opioid receptors. Accordingly, euphoric and analgesic effects vary (higher for methadone) and dysphoric effects are minor for buprenorphine (mediated by kappa-receptor antagonism). Moreover, the pharmaceutical form is different: drinkable solution for methadone and sublingual tablets for buprenorphine. Methadone has been shown to be significantly harder to be sold in prison than buprenorphine due to its physical characteristics, while the tablet form of buprenorphine may be particularly well-suited for sharing (15, 26). Previous studies concerning the practice of drug use in prison report intranasal administration of buprenorphine tablets by inmates (12, 23). Compared to intravenous administration they consider this practice safer and more practical. Additionally, it allows for a more rapid and intense onset of action (12). However, it should be taken into account that the illicit use of a drug, especially in a highly supervised setting such as prison, is always influenced by its availability.

Given that the illicit use of methadone and buprenorphine is highly prevalent in the prison population and that buprenorphine was found deriving from the community setting, we believe that the methods currently implemented to prevent and counter the entry of illicit drugs from the outside and the not intended use of drugs prescribed in prison (i.e., inspection by prison officers or by drug dogs on visitors and prisoners and random urine screening on prisoners) should be increased and extended. This could be achieved by identifying the people involved in the black market for substances outside and inside the prison, or by prescribing forms of medication that are less amenable to illicit use (e.g., buprenorphine-naloxone). Furthermore, our findings suggest a high number of inmates with untreated opioid use disorder. This emphasizes a need for a widespread implementation of OST in correctional facilities. Additionally, the use of illicitly obtained opioids during incarceration comes with an increased risk of overdosing (27). Hence, for individuals dying during incarceration or shortly after release, postmortem examination should routinely include systematic toxicological analyses.

Some limitations need to be considered for this study. The first limitation concerns the lack of clinical information in one of the prisons (Freiburg prison), which did not allow the authors to distinguish between prescribed and non-prescribed use of methadone in 19 cases. Another point may be that the biological matrix investigated (i.e., urine) only allowed for the detection of fairly recent drug consumption. Therefore, previous consumptions of OST drugs in individuals whose urine tested negative could not be revealed. This would have required multiple urine samples per inmate over an extended period of time or the additional collection of hair samples.

## 5. Conclusion

To the best of our knowledge, this is the first cross-sectional experimental study using toxicological analyses of urine samples for the detection of OST drugs in prisons and thus, being able to provide reliable information regarding illicit use of such drugs in jails.

The illicit use of methadone and buprenorphine was highly prevalent in the prison population investigated. Although the participation rate was high, the illicit use in the two prisons investigated could be even wider spread. Buprenorphine was the most common illicitly used drug by the study participants. It has also been found that in one of the prisons buprenorphine certainly was brought in from the outside.

Further cross-sectional experimental studies providing data on the prevalence of the illicit use of such drugs in prisons are needed to explore the trends of this phenomenon and counter it. Future research should also focus on performing toxicological analyses on biological samples which allow for a longer detectability of drug consumption (e.g., hair). Additionally, comprehensive toxicological analyses should be implemented to identify potentially life-threatening poly-drug use patterns.

## Data availability statement

The raw data supporting the conclusions of this article will be made available by the authors, without undue reservation.

## Ethics statement

The studies involving human participants were reviewed and approved by Ethics Committee - University of Freiburg. The patients/participants provided their written informed consent to participate in this study.

## Author contributions

AT-E, GF, and LH contributed to the conception and design of the study. GF organized the database and wrote the first draft of the manuscript. AT-E, LH, and VT wrote sections of the manuscript. All authors contributed to the manuscript revision, read, and approved the submitted version.

## Conflict of interest

The authors declare that the research was conducted in the absence of any commercial or financial relationships that could be construed as a potential conflict of interest.

## Publisher’s note

All claims expressed in this article are solely those of the authors and do not necessarily represent those of their affiliated organizations, or those of the publisher, the editors and the reviewers. Any product that may be evaluated in this article, or claim that may be made by its manufacturer, is not guaranteed or endorsed by the publisher.
